# Lipid-lowering therapy and low-density lipoprotein cholesterol goal attainment after acute coronary syndrome: a Danish population-based cohort study

**DOI:** 10.1186/s12872-020-01616-9

**Published:** 2020-07-13

**Authors:** Marie Skov Kristensen, Anders Green, Mads Nybo, Simone Møller Hede, Kristian Handberg Mikkelsen, Gunnar Gislason, Mogens Lytken Larsen, Annette Kjær Ersbøll

**Affiliations:** 1 National Institute of Public Health, University of Southern Denmark, Studiestræde 6, 1455 Copenhagen, Denmark; 2 Institute of Applied Economics and Health Research, N, 2200 Copenhagen, Denmark; 3 Open Patient data Explorative Network (OPEN), Odense University Hospital and University of Southern Denmark, Odense, Denmark; 4 Department of Clinical Biochemistry and Pharmacology, Odense University Hospital, Odense, Denmark; 5 Sanofi-Aventis Denmark A/S, Copenhagen, Denmark; 6 Department of Cardiology, The Cardiovascular Research Centre, Copenhagen University Hospital Herlev and Gentofte, Gentofte, Denmark; 7 Faculty of Health and Medical Sciences, Copenhagen University, Copenhagen, Denmark; 8 The Danish Heart Foundation, Copenhagen, Denmark; 9 Department of Cardiology, Aalborg University Hospital, Aalborg, Denmark

**Keywords:** Low-density lipoprotein cholesterol, LDL-C goal attainment, Dyslipidemia, Lipid-lowering therapy, Acute coronary syndrome, Population-based individual-level registers

## Abstract

**Background:**

Patients with acute coronary syndrome (ACS) are at high risk of recurrent cardiovascular (CV) event. The European guidelines recommend low-density lipoprotein cholesterol (LDL-C) levels < 1.8 mmol/L and early initiation of intensive lipid-lowering therapy (LLT) to reduce CV risk.

In order to reduce the risk of further cardiac events, the study aimed to evaluate LDL-C goal attainment and LLT intensity in an incident ACS population.

**Methods:**

A cohort study of patients with residency at Funen in Denmark at a first-ever ACS event registered within the period 2010–2015. Information on LLT use and LDL-C levels was extracted from national population registers and a Laboratory database at Odense University Hospital. Treatments and lipid patterns were evaluated during index hospitalization, at 6-month and 12-month follow-up.

**Results:**

Among 3040 patients with an LDL-C measurement during index hospitalization, 40.7 and 39.0% attained the recommended LDL-C target value (< 1.8 mmol/L) within 6- and 12-month follow-up, respectively. During 6- and 12-month follow-up, a total of 89.2% (20.2%) and 88.4% (29.7%) used LLT (intensive LLT). Of the intensive LLT users, 43.4 and 47.7% reached the LDL-C target value at 6- and 12-month follow-up. The frequency of lipid monitoring was low: 69.5, 77.7 and 53.6% in patients with a first-ever ACS during index hospitalization, 6- and 12-month follow-up, respectively.

**Conclusion:**

Using national health registers and laboratory data, a considerably gap was observed between treatment guidelines and clinical practice in the management of dyslipidemia leaving very high-risk patients without adequate lipid management strategy. Therefore, improved lipid management strategies aimed at reaching treatment targets are warranted.

## Background

Patients surviving an acute coronary syndrome (ACS) (i.e. diagnosis of acute myocardial infarction (AMI) or unstable angina (UA)) have an increased risk of recurrent cardiovascular (CV) events [1]. Early multidisciplinary cardiac rehabilitation to improve risk factors, e.g. smoking cessation, lifestyle advice and lipid profile modification is associated with reduced CV mortality [2].

In the management of dyslipidemia, it is well-established that lowering low-density lipoprotein cholesterol (LDL-C) concentration among very high-risk patients is the primary target to reduce the risk of CV events [3]. Until recently, the European clinical guidelines treatment goal for very high-risk patients was LDL-C <  1.8 mmol/L (< 70 mg/dL) or at least a 50% reduction in LDL-C if the baseline LDL-C was between 1.8–3.5 mmol/L [3]. According to current 2019 guidelines, the treatment goal is reduced to LDL-C <  1.4 mmol/L, which will cause even more attention on intensive LLT [4]. Today, ACS patients are already recommended to initiate high-intensity lipid-lowering therapy (LLT), mainly statins and/or combinational therapy, within the first 1–4 days of hospitalization [3, 5]. Yet, despite treatment with standard LLT, many European patients at very high risk of CV disease continue to have poorly controlled LDL-C levels and persistently high risk of recurrent CV events [6–8].

Statins are established as first-line LLT in ACS patients but novel agents for managing dyslipidemia are now available, such as proprotein convertase subtilisin-kexin type 9 (PCSK9) inhibitors. When guiding treatment decision for new LLTs in clinical practice it is valuable to gain insight into current treatment practice of dyslipidemia management.

Using Danish population-based health registers linked to clinical laboratory data, it is possible to provide information on lipid measurements and drug use at an individual level in a representative sample of the Danish population. In order to reduce the risk of further cardiac events, the aim of this study was to evaluate LDL-C goal attainment and the pattern of LLT use by intensity in patients with incident (first-ever) ACS.

## Methods

### Study design and setting

This is a population-based cohort study of the Funen population conducted in the study period January 1, 2010 to December 31, 2015. The population consists of approximately 0.5 million citizens (Q4 2018: 498,601 citizens) corresponding to 9% of the Danish population. The Region of Southern Denmark is a representative sample of the entire Danish population [9], which also applies to Funen [10]. The Danish healthcare system is tax-financed providing free access to treatment at hospitals and general practitioners. Most prescription drugs are covered by a reimbursement system applying for Danish citizens buying medication from a pharmacy [11].

### ACS and study population

Patients were eligible for inclusion if they 1) had experienced their first-ever qualifying ACS event within the study period (referred to as index hospitalization) and 2) had residency at Funen during index hospitalization. This comprised the *ACS population*.

Patients fulfilling the abovementioned criteria with at least one LDL-C measurement before or during index hospitalization (i.e., from 2 days before admission until discharge) identified in the laboratory database of Odense University Hospital, comprised the *study population*. Patients dying within 28 days after index hospitalization were excluded from the study.

ACS was identified in the Danish National Patient Register [12, 13] and defined as either a) primary diagnosis of AMI or b) a primary diagnosis of UA together with a primary procedure code of coronary angiography (CAG) during the same index hospitalization (see Additional file 1 for codes). Patients with a diagnosis of ACS in the period 1977–2009 were excluded to ascertain truly first-ever cases. In case of two CV events occurring at the same date, AMI events were registered and overruling any registration of UA with CAG. The positive predictive value for ACS diagnosis identified in the Danish National Patient Register is high (86.6%) [14].

### Data sources

Data sources comprised the Danish National Prescription Register, the Danish National Patient Register, and the Laboratory databases of Odense University Hospital. Individual-level linkage was facilitated by use of the unique civil registration number (CPR number) assigned to all individuals with permanent residence in Denmark at birth or immigration by the Danish Civil Registration System [15].

### Lipid measurements

Blood samples collected by general practitioners and at hospital wards at Funen were analyzed at hospital-based laboratories in the Funen County in the entire study period. Test results were stored in the Netlab Database until February 23, 2013 and in BCC from February 24, 2013 until the end of the study period. Data on lipid measurements were extracted from these databases with information on CPR number, test results of LDL-C, high-density lipoprotein cholesterol (HDL-C), total cholesterol and triglycerides, and the date of testing. Total cholesterol, HDL-C and triglycerides were measured in lithium-heparin plasma using an Architect c16000 analyzer (Abbott) with dedicated reagents, while LDL-C was calculated using Friedewald’s formula [16], when plasma triglycerides were ≤ 4 mmol/L (354 mg/dL), otherwise a direct plasma LDL-C measurement was performed with the same Abbott analyzer.

### Lipid-lowering therapy

Data on redeemed prescriptions of LLT were extracted from the Danish National Prescription Register [17], which contains data on all prescriptions redeemed at Danish pharmacies since 1995. Information on CPR number, the date of drug dispensing and administration of study drug using the Anatomical Therapeutic Chemical (ATC) classification codes were extracted from the register (see Additional file 1 for ATC codes). A total of 99% of the sale of lipid-modifying agents (ATC: C10) registered in the Danish National Prescription Registry was person-identifiable [18].

### Demographic and clinical characteristics

Information on the ACS population at index hospitalization on age, sex, cohabitation status and ethnicity was extracted from The Danish Civil Registration System. The mean annual disposable household income was extracted from Danish income registers at Statistics Denmark [19] 1 year prior to index hospitalization as a proxy measure for socioeconomic status. Information on comorbidity (chronic kidney disease and diabetes mellitus) was extracted five-year prior to index hospitalization. Chronic kidney disease was defined as either the first occurrence of a primary or secondary diagnosis or the first occurrence of a kidney transplantation identified in the Danish National Patient Register. Diabetes Mellitus Type 1 or 2 was defined as either the first occurrence of a primary or secondary diagnosis identified in the Danish National Patient Register or the first occurrence of a prescription redemption with antidiabetic drugs identified in the Danish National Prescription Register. See Additional file 1 for codes.

### Variable definitions

#### Low-density lipoprotein cholesterol

It is recommended to use LDL-C as the primary target to manage dyslipidemia when initiating and adjusting LLT [3]. LDL-C measurements were described *prior to index hospitalization* (i.e. from 545 days until 3 days before index hospitalization), *during index hospitalization* (i.e. from 2 days before index date until discharge) although a mean lowering of total cholesterol and LDL-C is well known between day 1 and days 2–4 [20], *at 6-month follow-up* (i.e. from discharge to 180 days after discharge), and *at 12-month follow-up* (i.e. from 181 days to 365 days after discharge) (Additional file 2). In case of more than one LDL-C measurement within the defined time windows, the LDL-C measurement closest to index hospitalization, 6-month and 12-month follow-up was used. For baseline characteristics, the ACS population was grouped into LDL-C <  1.8 mmol/L; LDL-C ≥ 1.8 mmol/L; and no LDL-C measurement.

#### Lipid-lowering therapy

The LLT prescription redemption pattern was described *prior to index hospitalization(i.e. the latest prescription of LLT redeemed from − 180 days until − 3 days before index hospitalization), during index hospitalization* (i.e. between index hospitalization until 30 days after discharge), *at 6-month.follow up* (i.e. between 31 days and 180 days after discharge), and *at 12-month follow-up* (i.e. between 181 days and 365 days after discharge) (Additional file 2).

LLT was categorized by intensity as “No LLT”, “Moderate LLT” or “Intensive LLT” [3, 21]. “Intensive LLT” was defined as having 1) a minimum of 2 prescription redemptions of one combination drug (statins in combination with Ezetimibe); or 2) a minimum of 2 prescription redemptions of statins (80 mg Simvastatin, 40–80 mg Atorvastatin or 20–40 mg Rosuvastatin); or 3) a minimum of 2 prescription redemptions of statins (all dose and types of statins) and one Ezetimibe prescription redemption; or 4) a minimum of 2 statins prescription redemptions and one other non-statin (i.e. not Ezetimibe, Evolocumab and Alirocumab) prescription redemption. “Moderate LLT” was defined as patients who were treated with LLT but were not eligible for the group of “Intensive LLT” or “No LLT”.

### Statistical analyses

Mean (standard deviations) or median (interquartile range) were reported for continuous variables and numbers and percentages for categorical variables. All patients were followed from inclusion (date of admission with first-ever ACS diagnosis) in 2010–2015 until death, end of study, or a new cardiac event, whichever came first. A new cardiac event was defined as a) primary or secondary diagnosis of AMI, b) a primary or secondary diagnosis of ischemic stroke, c) a primary diagnosis of UA together with a primary procedure code of CAG during the same index hospitalization, d) a primary diagnosis of stable angina together with a primary procedure code of CABG or PCI during the same index hospitalization, or e) a primary diagnosis or procedure code of peripheral arterial disease (see Additional file 1 for codes).

All analyses were conducted using SAS software version 9.4.

## Results

**Fig. 1:**
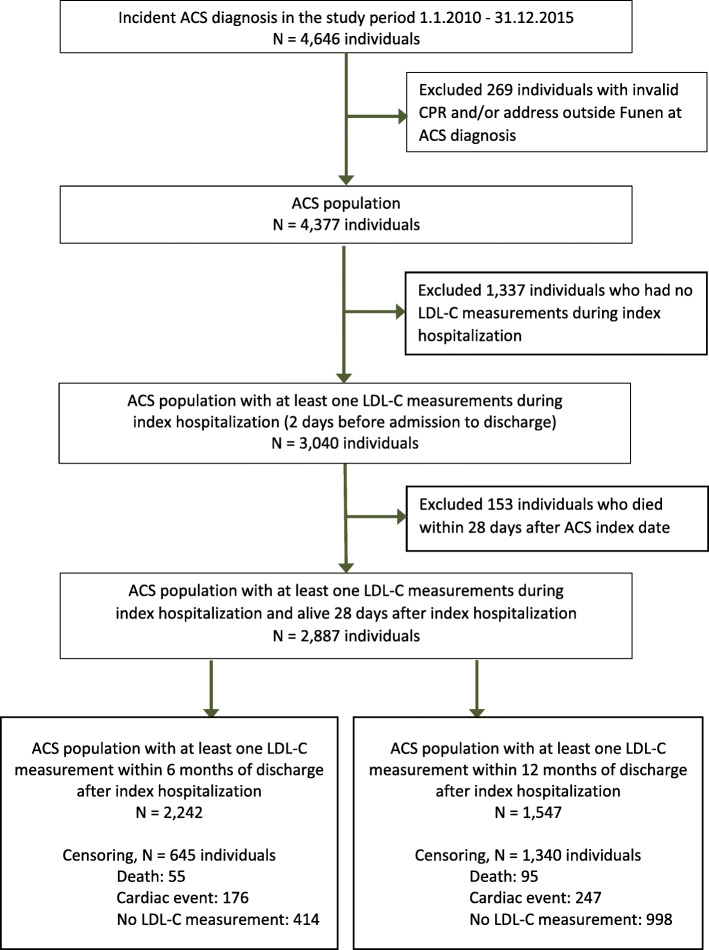
Flow diagram illustrating the construction of the ACS population and the study population (January 1, 2010 to December 31, 2015) based on data from nation-wide population registers. ACS, acute coronary syndrome; CPR, unique personal identification number; LDL-C, low-density lipoprotein cholesterol; Index hospitalization, hospitalization with incident ASC diagnosis

A total of 4646 patients were registered with a first-ever diagnosis of ACS within the study period (Fig. 1). Patients with an invalid CPR number or without residency at Funen at the year of index hospitalization (*n* = 269) were excluded. Of the remaining 4377 patients (i.e., ACS population), 3040 (69.5%) had at least one LDL-C measurement during index hospitalization (Table 1). Of those, 153 patients died within 28 days after index hospitalization leaving 2887 patients for inclusion as the study population in the analysis (Fig. 1).

**Table 1:** Baseline characteristics of the ACS population stratified by low-density lipoprotein cholesterol (LDL-C) (LDL-C < 1.8 mmol/L, LDL-C ≥ 1.8 mmol/L, no measurement) given by number and percentage (N, %) if nothing else is indicated (1 January 2010–31 December 2015)

Variable		ACS population, *N* = 4377		
	N (%)	LDL-C (mmol/L) during index hospitalization^a^		
	Total	< 1.8	≥ 1.8	No
Overall	4377	322 (7.4)	2718 **(**62.1)	1337 **(**30.5)
ACS at index date				
AMI	3891 (88.9)	275 (85.4)	2530 (93.1)	1086 (81.2)
UA + CAG	486 (11.1)	47 (14.6)	188 (6.9)	251 (18.8
Age, mean ± SD	67.8 ± 13.6	70.4 ± 11.9	65.5 ± 13.4	71.8 ± 13.3
Age groups				
< 40	73 (1.7)	5 (1.6)	58 (2.1)	10 (0.7)
40–49	366 (8.4)	8 (2.5)	295 (10.9)	63 (4.7)
50–59	775 (17.7)	42 (13.0)	558 (20.5)	175 (13.1)
60–69	1126 (25.7)	88 (27.3)	718 (26.4)	320 (23.9)
70–79	1067 (24.4)	103 (32.0)	622 (22.9)	342 (25.6)
≥ 80	970 (22.2)	76 (23.6)	467 (17.2)	427 (31.9)
Gender				
Male	2787 (63.7)	219 (68.0)	1816 (66.8)	752 (56.2)
Female	1590 (36.3)	103 (32.0)	902 (33.2)	585 (43.8)
Cohabitation				
Yes	2490 (56.9)	191 (59.3)	1611 (59.3)	688 (51.5)
No	1887 (43.1)	131 (40.7)	1107 (40.7)	649 (48.5)
Ethnicity				
Denmark	4101 (93.7)	305 (94.7)	2533 (93.2)	1263 (94.5)
Western	106 (2.4)	< 5	70 (2.6)	< 35
Non-western	170 (3.9)	< 15	115 (4.2)	< 45
Comorbidity				
Diabetes mellitus	735 (16.8)	128 (39.8)	324 (11.9)	283 (21.2)
Chronic Kidney disease	183 (4.2)	22 (6.8)	82 (3.0)	79 (5.9)
Socioeconomic position				
< 100,000 DKK	432 (9.9)	37 (11.5)	257 (9.5)	138 (10.3)
100,000 – 299,999	3497 (79.9)	261 (81.1)	2134 (78.5)	1102 (82.4)
≥ 300,000	448 (10.2)	24 (7.5)	327 (12.0)	97 (7.3)
LLT^b^ before or during index hospitalization				
Statins	1338 (30.6)	233 (72.4)	608 (22.4)	497 (37.2)
Ezetimibe	21 (0.5)	< 5	16 (0.6)	< 5
Other non-statins	13 (0.3)	< 5	6 (1.9)	< 10
Combinational treatment	15 (0.3)	< 10	6 (1.9)	< 5
No treatment	2990 (68.3)	79 (24.5)	2082 (76.6)	829 (62.0)
LLT intensity^b^ before or during index hospitalization				
No LLT	2990 (68.3)	79 (24.5)	2082 (76.6)	829 (62.0)
Moderate LLT^c^	1262 (28.8)	220 (68.3)	579 (21.3)	463 (34.6)
Intensive LLT^d^	125 (2.9)	23 (7.1)	57 (2.1)	45 (3.4)
Lipid measurements before index hospitalization^e^, N (%), mean ± SD				
LDL-C	2967 (67.8) 2.96 ± 1.12	272 (84.5) 1.86 ± 0.68	1733 (63.8) 3.22 ± 1.04	962 (72.0) 2.80 ± 1.14
HDL-C	2968 (67.8) 1.33 ± 0.43	272 (84.5) 1.32 ± 0.48	1734 (63.8) 1.31 ± 0.39	962 (72.0) 1.37 ± 0.47
Total cholesterol	2995 (68.4) 5.07 ± 1.28	272 (84.5) 3.97 ± 0.91	1750 (64.4) 5.33 ± 1.20	973 (72.8) 4.92 ± 1.31
Triglycerides	2968 (67.8) 1.80 ± 1.23	272 (84.5) 1.84 ± 1.43	1734 (63.8) 1.86 ± 1.28	962 (72.0) 1.69 ± 1.07

*ACS* Acute coronary syndrome; *AMI* Acute myocardial infarction; *UA* unstable angina; *CAG* Coronary angiography; *LLT* Lipid-lowering treatment; *LDL-C* Low-density lipoprotein cholesterol; *HDL-C* high-density lipoprotein cholesterol; *SD* Standard deviation

^a^ Index hospitalization from 2 days before index date until discharge

^b^ LLT initiated before or during index hospitalization: LLT redeemed −180 prior admission to 30 days

after discharge

^c^ Patients who are treated with LLT, but who are not eligible for the group of “intensive treatment” or “no LLT” treatment

^d^ Intensive LLT is defined by 1) A minimum of 2 prescription redemptions of one combination drug; *OR* 2) A minimum of 2 statins prescription redemptions; *OR* 3) A minimum of 2 statin prescription redemptions and one ezetimibe prescription redemption; *OR* 4) A minimum of 2 statin prescription redemptions and one other non-statin (i.e. not ezetimibe) prescription redemption

^e^ Lipid measurements prior index hospitalization: from −545 days to −3 days before index hospitalization

### Baseline demographic and clinical characteristics

During index hospitalization, 1337 (30.5%) patients had no LDL-C measurement. Among patients with an LDL-C measurement during index hospitalization, LDL-C was above target value (1.8 mmol/L) for 89.4% of the patients (2718 / (2718 + 322)). The most common index hospitalization was AMI (88.9%). Patients with no LDL-C measurement during index hospitalization (71.8 ± 13.3 years) were on average 1.4 and 6.3 years older than patients with an LDL-C <  1.8 mmol/L (70.4 ± 11.9 years) and LDL-C ≥ 1.8 mmol/L (65.5 ± 13.3 years), respectively. Further, more females (43.8% vs. 33.1%) and fewer cohabitants (51.5% vs. 59.3%) were represented among patients with no LDL-C measurement compared with patients with an LDL-C measurement (Table 1).

### LLT utilization and LDL-C levels prior to index hospitalization

Less than a third (31.7%) of the ACS population were registered with an LLT prescription redemption before index hospitalization. Among patients with an LDL-C < 1.8 mmol/L during index hospitalization, 75.4% were in LLT prior to index hospitalization including 7.1% being intensive users.

Among patients with an LDL-C ≥ 1.8 mmol/L, 23.4% were treated with LLT including 2.1% in intensive LLT. The preferred LLT was statins. Prior to index hospitalization, 84.5% of patients with an LDL-C < 1.8 mmol/L during index hospitalization, 63.8% of patients with an LDL-C ≥ 1.8 mmol/L and 72.0% of patients with no LDL-C had obtained a lipid profile. The mean (SD) LDL-C prior to index hospitalization was distributed as follows according to levels of LDL-C during index hospitalization: LDL-C < 1.8 mmol/L (1.86 ± 0.68); LDL-C ≥ 1.8 mmol/L (3.22 ± 1.04); and no measurement (2.80 ± 1.14) (Table 1).

### LLT utilization and LDL-C goal attainment at follow-up

Among the ACS population with at least one LDL-C measurement during index hospitalization and alive 28 days after index hospitalization (*n* = 2887), 2242 (77.7%) had at least one LDL-C measurement registered at 6-month follow-up and 1547 (53.6%) had at least one LDL-C measurement registered at 12-month follow-up (Fig. 1).

**Fig. 2:**
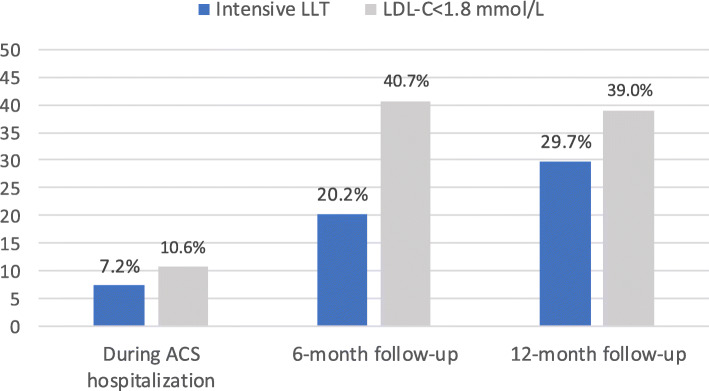
Distribution of intensive lipid-lowering treatment (LLT) and low-density lipoprotein cholesterol (LDL-C) goal ascertainment (LDL-C < 1.8 mmol/L) in patients diagnosed with acute coronary syndrome (ACS) with an LDL-C measurement during ACS hospitalization (*N* = 3040), 6-month (*N* = 2242) and 12-month follow-up (*N* = 1547)

During hospitalization, only 7.2% of the patients diagnosed with ACS initiated or continued intensive LLT treatment. In total, 76.4% of the patients initiated or continued moderate LLT treatment. Although the proportion of ACS patients treated with intensive LLT increased during follow-up, only a minority of the patients were treated with intensive LLT as recommended in guidelines (20.2 and 29.7% at 6- and 12-month follow-up). During follow-up, LDL-C goal ascertainment (LDL-C < 1.8 mmol/L) increased from 10.6% during index hospitalization to 40.7 and 39.0% during 6- and 12-month follow-up leaving about 60% of the ACS patients with lack of goal ascertainment (Fig. 2).

### 6-month follow-up

Among those with a 6-month LDL-C measurement, 912 patients (40.7%) attained LDL-C < 1.8 mmol/L. A total of 2069 (92.3%) used LLT (8.1% intensive LLT) before or during index hospitalization. Among those with an LDL-C < 1.8 mmol/L at 6-month follow-up, 42.4 and 42.5% were treated with moderate or intensive LLT (Table 2).

**Table 2:** Distribution of the study population by lipid-lowering therapy (LLT) intensity initiated during hospitalization, at 6-month and 12-month follow-up, and low-density lipoprotein cholesterol (LDL-C) goal attainment at 6-month and 12-month follow-up (1 January 2010–31 December 2015)

		LDL-C (mmol/L)					
		6-month follow-up^d^ (*N* = 2242)			12-month follow-up^e^ (*N* = 1547)		
**LLT**		Total	< 1.8	≥1.8	Total	< 1.8	≥1.8
Before or during index hospitalization^a^		2242	912 (40.7)	1330 (59.3)	1547	604 (39.0)	943 (61.0)
None		173 (7.7)	34 (19.7)	139 (80.3)	122 (7.9)	25 (20.5)	97 (79.5)
Moderate^b^		1888 (84.2)	801 (42.4)	1087 (57.6)	1282 (82.9)	519 (40.5)	763 (59.5)
Intensive^c^		181 (8.1)	77 (42.5)	104 (57.5)	143 (9.2)	60 (42.0)	83 (58.0)
**LLT**							
Before or during index hospitalization^a^	6-month follow-up						
None	None	120/173 (69.4)	17/120 (14.2)	103/120 (85.8)	89/122 (73.0)	11/89 12.4)	78/89 (87.6)
	Moderate^b^	46/173 (26.6)	< 15	< 35	28/122 (23.0)	< 15	< 20
	Intensive^c^	7/173 (4.0)	< 7	< 7	5/122 (4.1)	< 5	< 5
Yes	None	123/2069 (5.9)	41/123 (33.3)	82/123 (66.7)	72/1425 (5.1)	19/72 (26.4)	53/72 (73.6)
	Moderate^b^	1501/2069 (72.5)	644/1501 (42.9)	857/1501 (57.1)	1041/1425 (73.1)	425/1041 (40.8)	616/1041 (59.2)
	Intensive^c^	445/2069 (21.5)	193/445 (43.4)	252/445 (56.6)	312/1425 (21.9)	135/312 (43.3)	177/312 (56.7)
**LLT**							
6-month follow-up	12-month follow-up						
None	None	–	–	–	113/161 (70.2)	15/113 (13.3)	98/113 (86.7)
	Moderate^b^	–	–	–	38/161 (23.6)	< 15	< 30
	Intensive^c^	–	–	–	10/161 (6.2)	< 5	< 10
Yes	None	–	–	–	66/1386 (4.8)	13/66 (19.7)	53/66 (80.3)
	Moderate^b^	–	–	–	871/1386 (62.8)	347/871 (39.8)	524/871 (60.2)
	Intensive^c^	–	–	–	449/1386 (32.4)	214/449 (47.7)	235/449 (52.3)

^a^ LLT initiated before or during index hospitalization: LLT redeemed −180 before admission to 30 days

after discharge

^b^ Patients who are treated with LLT, but who are not eligible for the group of “intensive treatment” or “no LLT” treatment

^c^ Intensive LLT is defined by 1) A minimum of 2 prescription redemptions of one combination drug; *OR* 2) A minimum of 2 statins prescription redemptions; *OR* 3) A minimum of 2 statin prescription redemptions and one ezetimibe prescription redemption; *OR* 4) A minimum of 2 statin prescription redemptions and one other non-statin (i.e. not ezetimibe) prescription redemption

^d^ LDL-C at 6-month follow-up: LDL-C between discharge and 180 days post discharge from hospital with ACS

^e^ LDL-C at 12-month follow-up: LDL-C between 181 and 365-days post discharge from hospital with ACS

Among those who continued using LLT at 6-month follow-up (94.0%, including 21.5% intensive users), the proportion of moderate and intensive LLT users reaching LDL-C < 1.8 mmol/L remained unchanged (42.9 and 43.4%). Among those who used LLT before or during index hospitalization, 5.9% had discontinued LLT during 6-month follow-up, among these 66.7% with an LDL-C ≥ 1.8 mmol/L (Table 2).

### 12-month follow-up

Among those with a 12-month LDL-C measurement, 604 patients (39.0%) had achieved LDL-C. < 1.8 mmol/L. Among patients using LLT at 6-month follow-up, 95.2% continued using LLT at 12-month follow-up (62.8% with moderate LLT and 32.4% with intensive LLT). Of the moderate and intensive LLT users, 39.8 and 47.7% had achieved LDL-C < 1.8 mmol/L, respectively.

Among patients with LLT during 6-month follow-up and with moderate or intensive LLT at 12-month follow-up, a total of 60.2 and 52.3% had an LDL-C ≥ 1.8 mmol/L at 12-month follow-up. For comparison, among patients with or without LLT during 6-month follow-up and no LLT at 12-month follow-up, a total of 80.3 and 86.7% had an LDL-C ≥ 1.8 mmol/L at 12-month follow-up (Table 2).

### Stratified by comorbidity (diabetes mellitus and/or chronic kidney disease)

Among the ACS population with at least one LDL-C measurement during index hospitalization and alive 28 days after index hospitalization (*n* = 2887), a total of 16.5% had diabetes mellitus and/or chronic kidney disease at inclusion. At index hospitalization, 24.2% of the patients with a comorbidity had an LDL-C < 1.8 mmol/L as compared to 7.8% among patient without a comorbidity at index hospitalization (Table 3). A larger proportion of patients with comorbidity obtained LDL-C goal attainment during 6- and 12-month follow-up as compared to patients without a comorbidity (49.7% versus 38.7% at 6-month follow-up and 51.8% versus 36.5% at 12-month follow-up).

**Table 3:** Low-density lipoprotein cholesterol (LDL-C) goal attainment of the ACS population with at least one LDL-C measurements during index hospitalization and alive 28 days after index hospitalization (*N* = 2887) stratified by baseline comorbidity (diabetes mellitus and/or chronic kidney disease). LDL-C level (LDL-C < 1.8 mmol/L, LDL-C ≥ 1.8 mmol/L, no measurement) given by number and percentage (N, %) if nothing else is indicated (1 January 2010–31 December 2015)

Comorbidity	Total	ACS population, *N* = 2887		
		LDL-C (mmol/L)		
		< 1.8	≥ 1.8	No
**Comorbidity at inclusion (diabetes mellitus and/or chronic kidney disease)**				
LDL-C measurement at				
Index hospitalization	477	104 (24.2)	325 (75.8)	48
6-month follow-up	417	168 (49.7)	170 (50.3)	79
12-month follow-up	381	128 (51.8)	119 (48.2)	134
**No comorbidity at inclusion**				
LDL-C measurement at				
Index hospitalization	2410	115 (7.8)	1350 (92.2)	945
6-month follow-up	2239	684 (38.7)	1085 (61.3)	470
12-month follow-up	2164	455 (36.5)	793 (63.5)	916

*ACS* Acute coronary syndrome; *LDL-C* Low-density lipoprotein cholesterol

## Discussion

ACS patients are recommended to initiate intensive LLT. However, using complete population-based health registers linked to clinical laboratory data, this study provided important insight into management of dyslipidemia among ACS patients in Denmark and highlights a considerable gap between recommendation in treatment guidelines (2016) [3] and clinical practice. We found that the LDL-C goal attainment was low at 6- and 12-month follow-up, respectively. Most of the study population used LLT, but only a minority used intensive LLT as recommended throughout follow-up. Improvement in LLT management in this high-risk population is needed. A larger proportion of the ACS patients with a comorbidity had LDL-C < 1.8 mmol/L at index hospitalization and a larger proportion obtained LDL-C goal attainment at 6- and 12-month follow-up.

### LDL-C goal attainment

According to treatment guidelines [3] it is recommended that patients at very high risk of a CV event should achieve LDL-C < 1.8 mmol/L [3]. Moreover, the national quality indicators of cardiac rehabilitation in Denmark states that ≥70% of patients with ischemic heart disease should reach the LDL-C target value < 1.8 mmol/L at the end of a rehabilitation program [22]. However, in this study the LDL-C goal attainment was low with less than half of the study population achieving LDL-C < 1.8 mmol/L at 6- and 12-month follow-up. Treatment with LLT increased the likelihood of reaching the target value of LDL-C < 1.8 mmol/L compared with no LLT use. Yet, the proportion reaching the target value did not differ substantially between moderate and intensive LLT users during index hospitalization and 6-month follow-up, but the likelihood of achieving LDL-C < 1.8 mmol/L at 12-month follow-up was higher among intensive LLT users compared with moderate LLT users. Similar findings were demonstrated in the Netherlands among 2431 hospitalized ACS patients where no substantial difference in the proportion of patients achieving LDL-C goal was seen irrespective of treatment with statins plus ezetimibe or statin as monotherapy [7]. In general, poor LDL-C goal attainment have been reported in several European studies among patients hospitalized for ACS (18.9–55%) [7, 8, 23–25]. It is well-documented that lowering LDL-C in very-high risk patients have direct cardiovascular benefits. A meta-analysis of 26 randomized trials demonstrated a decrease of major CV events by 22% for each mmol/L reduction in LDL cholesterol [26]. Attending a multidisciplinary cardiac rehabilitation program targeting improvement in LDL-C levels is associated with reduced risk of morbidity and mortality in patients with ACS [3]. Despite this, it is documented that cardiac rehabilitation is underused in Europe and the US [27] including Denmark with only 61% of ACS patients attending a cardiac rehabilitation program [28].

### LLT utilization

We observed that only a third were on LLT prior to first-ever ACS diagnosis indicating an unmet need for identifying patients with a high-risk cardiovascular profile who potentially may benefit from primary prevention in terms of early initiation of LLT [29]. Yet, a high rate of LLT utilization was observed among the study population after being diagnosed with ACS. A similar high LLT use among ACS patients has been reported in previously studies (at admission: 90.7 and 96.6%, at 120-days follow-up: 85.9 and 96.6%) [24, 25]. Despite this, we found that only few were treated with intensive LLT throughout the study period, which is in discordance with the treatment guidelines recommending initiation of intensive LLT immediately regardless of baseline cholesterol levels until treatment goal attainment [3]. Evidence has shown that, intensive LLT reduces the risk of non-fatal and fatal cardiovascular events to a greater extent than low-to-moderate LLT [30].

Our result is consistent with existing studies, demonstrating underutilization of intensive LLT among very high-risk patients in several European countries. For example, a large study (EUROSPIRE IV) conducted among 24 European countries found that 37.6% were discharged for coronary artery disease with high intensive statin decreasing to 32.7% high-intensive users at 6-month follow-up [31].

Another important finding is that a large proportion of intensive users failed to reach LDL-C treatment goal. In this study, intensive LLT was mainly driven by statins with a smaller proportion using combinational therapy e.g. statins plus ezetimibe. As prescribed in the treatment guidelines [3], patients with statin intolerance should be offered add-on treatment in terms of Ezetimibe and in patients not reaching the recommended LDL-C target despite maximally tolerated dose PCSK9 inhibitors might be considered. Our finding of a low proportion of patients reaching LDL-C target level align with the findings of a recent cohort study from another part of Denmark [32]. However, the two studies differ on two important aspects, the study population and aim of the studies. Sundbøll et al. [32] included a population of individuals with a prevalent atherosclerotic cardiovascular disease (ASCVD), having an LDL-C ≥ 1.8 mmol/L and using LLT (statins or ezetimibe). The aim of their study was to estimate cardiovascular event rates. They also reported patterns of LLT and LDL-C levels during follow-up. In the present study, we included a population of patients with a first-ever incident ACS. In order to reduce the risk of further cardiac events, LLT initiation is very important in order to obtain LDL-C goal attainment with LDL-C reduced to a value below 1.8 mmol/L. Therefore, our aim was to evaluate and examine LDL-C goal attainment and patterns of LLT use in this population of patients presented with an ACS for the first time ever.

Current registers in Denmark do not provide information on the clinician’s rationale for choosing a certain dose or type of LLT. One explanation for not choosing intensive LLT may be clinicians’ lack of knowledge of the advantage of intensive LLT [33]. Another explanation may be safety issues in older persons are of special concern when prescribing LLT due to comorbidities and polypharmacy [33]. In this study, three out of four patients were ≥ 60 years of age. According to treatment guidelines, older persons are recommended to initiate LLT in a low dose due to safety issues following up-titration to treatment goal [3]. Yet, these recommendations are not consistent with guidelines for dyslipidemia in ACS patients which recommend intensive LLT in all patients presenting with ACS as soon as possible during admission. As suggested elsewhere, alternative guidelines for older persons with ACS aiming to address concerns for possible medication interaction when treated with LLT may be helpful in clinician’s decision making ensuring older people optimal dyslipidemia treatment [33].

### Monitoring of LDL-C levels

We observed low frequencies of lipid monitoring among the study population. During index hospitalization less than three out of four had lipids measured, which is in discordance with treatment guidelines recommending all patients to obtain lipid profile during admission. Further, when ACS patients are discharged from the hospital their general practitioners are informed about follow-up and guidelines recommend re-evaluating LDL-C 4–6 weeks post index hospitalization to ensure safety issues and to adapt LLT dose. In this study, nearly three out of four underwent lipid testing between discharge for ACS and 180 days after discharge. Moreover, only half of the study population underwent LDL-C measurement between 181 days and 365 days post discharge. There is a need to focus on follow-up and reaching targets goals in secondary prevention management. Ensuring bilateral communication between hospitals and primary care that manage patients after discharge might improve this.

### Study limitations

Limitations include no information on the actual consumption of drugs, no information on drugs dispensed directly to the patients in the hospital, and no reason for drug discontinuation. The study design is an observational retrospective cohort study. However, although a retrospective nature of the study (due to using already collected data), data are prospectively collected in administrative registers including hospital contacts and diagnoses, prescription redemptions, and a laboratory database with test results of blood samples for the study population is used. Advantages of using these data sources include a large sample size and no selection bias. However, data are limited to the information and variables entered in the registers. Therefore, no information about other important variables such as lifestyle habits including smoking, diet and exercise was available. Furthermore, no data with information about the incidence of arterial hypertension were available for the study. Finally, inclusion of data from all laboratories in Denmark would have strengthened the study; however, data from one centralized laboratory can also be beneficial as it ensures more homogenous data with 100% comparability without any analytical differences.

## Conclusions

In this population-based cohort study, we found a considerably gap between the European treatment guidelines and current clinical practice in patients with a first-ever ACS diagnosis. By use of health registers and laboratory data it is possible to monitor clinical practice and these findings reflect a great potential to improve LDL-C goal attainment through optimizing current treatment practice aiming to reduce risk of a recurrent CV event.

## Supplementary information

**Additional file 1.**

**Additional file 2.**

## Data Availability

The datasets generated during and/or analysed during the current study are not publicly available due to data privacy regulation by Statistics Denmark.

## Abbreviations

ACS: Acute coronary syndrome
AMI: Acute myocardial infarction
ATC: Anatomical therapeutic chemical
CAG: Coronary angiography
CPR number: Civil registration number
CV: Cardiovascular
HDL-C: High-density lipoprotein cholesterol
LDL-C: Low-density lipoprotein cholesterol
LLT: Lipid-lowering therapy
PCSK9: Proprotein convertase subtilisin-kexin type 9
SD: Standard deviation
UA: Unstable angina

## References

[1] Abu-Assi E, López-López A, González-Salvado V (2016). The risk of cardiovascular events after an acute coronary event remains high, especially during the first year, despite revascularization. Rev Esp Cardiol Engl Ed.

[2] Anderson L, Oldridge N, Thompson DR (2016). Exercise-based cardiac rehabilitation for coronary heart disease. J Am Coll Cardiol.

[3] Catapano AL, Graham I, De Backer G (2016). 2016 ESC/EAS guidelines for the Management of Dyslipidaemias. Eur Heart J.

[4] Mach F, Baigent C, Catapano AL (2020). 2019 ESC/EAS guidelines for the Management of Dyslipidaemias. Eur Heart J.

[5] Ray KK, Cannon CP, McCabe CH (2005). Early and late benefits of high-dose atorvastatin in patients with acute coronary syndromes. J Am Coll Cardiol.

[6] März W, Dippel F-W, Theobald K, Gorcyca K, Iorga ŞR, Ansell D (2018). Utilization of lipid-modifying therapy and low-density lipoprotein cholesterol goal attainment in patients at high and very-high cardiovascular risk: real-world evidence from Germany. Atherosclerosis.

[7] Kuiper JG, Sanchez RJ, Houben E (2017). Use of Lipid-modifying Therapy and LDL-C Goal Attainment in a High-Cardiovascular-Risk Population in the Netherlands. Clin Ther.

[8] Gitt AK, Lautsch D, Ferrières J (2017). Cholesterol target value attainment and lipid-lowering therapy in patients with stable or acute coronary heart disease: results from the dyslipidemia international study II. Atherosclerosis.

[9] Henriksen DP, Rasmussen L, Hansen MR, Hallas J, Pottegård A (2015). Comparison of the five Danish regions regarding demographic characteristics, healthcare utilization, and medication use—a descriptive cross-sectional study Dalal K, editor. PLoS One.

[10] Anon. Statistics Denmark. StatBank Denmark. https://statistikbanken.dk. Accessed 7 Mar 2015.

[11] Anon. Healthcare in Denmark: an overview. Ministry of Health; 2016.

[12] Lynge E, Sandegaard JL, Rebolj M (2011). The Danish National Patient Register. Scand J Public Health.

[13] Nordisk Medicinal-Statistisk Komité (2010). NOMESCO classification of surgical procedures.

[14] Bork CS, Al-Zuhairi KS, Hansen SM, Delekta J, Joensen AM (2017). Accuracy of angina pectoris and acute coronary syndrome in the Danish National Patient Register. Dan Med J.

[15] Pedersen CB (2011). The Danish civil registration system. Scand J Public Health.

[16] Friedewald WT, Levy RI, Fredrickson DS (1972). Estimation of the concentration of low-density lipoprotein cholesterol in plasma, without use of the preparative ultracentrifuge. Clin Chem.

[17] Kildemoes HW, Sørensen HT, Hallas J (2011). The Danish National Prescription Registry. Scand J Public Health.

[18] Schmidt M, Hallas J, Laursen M, Friis S (2016). Data resource profile: Danish online drug use statistics (MEDSTAT). Int J Epidemiol.

[19] Baadsgaard M, Quitzau J (2011). Danish registers on personal income and transfer payments. Scand J Public Health.

[20] Barth JH, Jackson BM, Farrin AJ (2010). Change in serum lipids after acute coronary syndromes: secondary analysis of SPACE ROCKET study data and a comparative literature review. Clin Chem.

[21] Cannon CP, Braunwald E, CH MC, Rader DJ, Rouleau JL, Belder R, Joyal SV, Hill KA, Pfeffer MA, Skene AM (2004). For the pravastatin or atorvastatin evaluation and infection therapy-thrombolysis in myocardial infarction 22 investigators. Intensive versus moderate lipid lowering with statins after acute coronary syndromes. N Engl J Med.

[22] Anon. Indicators and Standards for Danish Cardiac Rehabilitation Database for Patients Initiating a Cardiac Rehabilitation, 2018. https://www.rkkp.dk/om-rkkp/de-kliniske-kvalitetsdatabaser/hjerterehabilitering. Accessed 1 Apr 2019.

[23] Gitt AK, Lautsch D, Ferrières J (2018). Contemporary data on treatment practices for low-density lipoprotein cholesterol in 3867 patients who had suffered an acute coronary syndrome across the world. Data Brief.

[24] Ferrières J, Rouyer MV, Lautsch D (2017). Suboptimal achievement of low-density lipoprotein cholesterol targets in French patients with coronary heart disease. Contemporary data from the DYSIS II ACS/CHD study. Arch Cardiovasc Dis.

[25] Gitt AK, Rieber J, Hambrecht R, et al. Do acute coronary events affect lipid management and cholesterol goal attainment in Germany?: results from the dyslipidemia international study II. Wien Klin Wochenschr 2018. Available at: http://link.springer.com/10.1007/s00508-018-1375-3. . 10.1007/s00508-018-1375-3 PMC6290720 30178071

[26] Baigent C, Blackwell L, Cholesterol Treatment Trialists’ (CTT) Collaboration (2010). Efficacy and safety of more intensive lowering of LDL cholesterol: a meta-analysis of data from 170,000 participants in 26 randomised trials. Lancet.

[27] Piepoli MF, Corrà U, Benzer W (2010). Secondary prevention through cardiac rehabilitation: from knowledge to implementation. A position paper from the cardiac rehabilitation section of the European Association of Cardiovascular Prevention and Rehabilitation. Eur J Cardiovasc Prev Rehabil.

[28] Graversen CB, Eichhorst R, Ravn L, Christiansen SSR, Johansen MB, Larsen ML (2017). Social inequality and barriers to cardiac rehabilitation in the rehab-north register. Scand Cardiovasc J.

[29] Brugts JJ, Yetgin T, Hoeks SE (2009). The benefits of statins in people without established cardiovascular disease but with cardiovascular risk factors: meta-analysis of randomised controlled trials. BMJ.

[30] Cannon CP, Braunwald E, McCabe CH (2004). Intensive versus moderate lipid lowering with statins after acute coronary syndromes. N Engl J Med.

[31] Reiner Ž, De Backer G, Fras Z (2016). Lipid lowering drug therapy in patients with coronary heart disease from 24 European countries – Findings from the EUROASPIRE IV survey. Atherosclerosis.

[32] Sundbøll J, Larsen AP, Veres K, Adelborg K, Sørensen HT (2019). Cardiovascular event rates and trajectories of LDL-cholesterol levels and lipid-lowering therapy in patients with atherosclerotic cardiovascular disease: a population-based cohort study. Thromb Res.

[33] Hirsh BJ, Smilowitz NR, Rosenson RS, Fuster V, Sperling LS (2015). Utilization of and adherence to guideline-recommended lipid-lowering therapy after acute coronary syndrome. J Am Coll Cardiol.

[34] Thygesen LC, Daasnes C, Thaulow I, Brønnum-Hansen H (2011). Introduction to Danish (nationwide) registers on health and social issues: structure, access, legislation, and archiving. Scand J Public Health.

