# Supplementary material for: Lipid-lowering therapy and low-density lipoprotein cholesterol goal attainment after acute coronary syndrome: a Danish population-based cohort study
Source: BMC Cardiovasc Disord. 2020 Jul 13;20:336. doi: 10.1186/s12872-020-01616-9 (PMC7359510; doi:10.1186/s12872-020-01616-9)
Supplement: Supplementary file 1 — Additional file 1. [file 12872_2020_1616_MOESM1_ESM.docx]

**Table 3** Codes for diagnoses, surgeries and drugs

| **Variable** | **Register and coding system** | **Codes** |
| --- | --- | --- |
| Acute myocardial infarction (AMI) | The Danish National Patient Register  (ICD codes) | 410* (ICD-8)  I21*, I22*, I23* (ICD-10) |
| Unstable Angina (UA) | The Danish National Patient Register  (ICD codes) | 411* (ICD-8)  I200* (ICD-10) |
| Ischemic stroke (IS) | The Danish National Patient Register  (ICD codes) | I63*, I64* (ICD-10) |
| Stable Angina (SA) | The Danish National Patient Register  (ICD codes) | I201-I209 (ICD-10) |
| Peripheral arterial disease (PAD) | The Danish National Patient Register  (ICD codes) | I65*, I66*, I70*- I74*, I77* (ICD-10) |
| Coronary angiography (CAG) | The Danish National Patient Register (NOMESCO) | UXAC85 |
| Coronary bypass operation (CABG) | The Danish National Patient Register (NOMESCO) | KFNA*-KFNE*, KFNH20 |
| Percutaneous transluminal coronary intervention (PCI) | The Danish National Patient Register (NOMESCO) | KFNG00-KFNG12, KFNG30 |
| Chronic kidney disease | The Danish National Patient Register  (ICD codes) | 581*-584* (ICD-8)  N02*-N08*, N11*-N14*, N158*-N160*, N162*-N165*, N168, N18*-N19*, Z940, Z992 E102, E112, E122, E132, E142, I12*-I13*, Q61*, M300, M313, M319, M321B, N26* (ICD-10) |
|  | The Danish National Patient Register (NOMESCO) | 94300, 94340, 5748*  BJFD2*, KKAS* (NOMESCO codes) |
| Diabetes mellitus | The Danish National Patient Register  (ICD codes) | 249.00*-250.09* (ICD-8)  E10.0-E14.9* (ICD-10) |
|  | The Danish National Prescription Registry (ATC codes) | A10A*, A10B*, C10AB04* |
| Statins | The Danish National Prescription Registry (ATC codes) | C10AA01- C10AA07 |
| Ezetimibe |  | C10AX09, C10AX13, C10AX14 |
| Other non-statins |  | C10AB01, C10AB02, C10AB04, C10AC01, C10AC02, C10AC04, C10AD06, C10AD52 |
| Combinational therapy |  | C10BA02, C10BA05 |

*means inclusion of all underlying codes. ICD codes: International Classification of Diseases (ICD) versions 8 and 10, NOMESCO codes: the Nordic Medical Statistics Committees Classification of Surgical Procedures
