# Supplementary material for: Lipid-lowering therapy and low-density lipoprotein cholesterol goal attainment after acute coronary syndrome: a Danish population-based cohort study
Source: BMC Cardiovasc Disord. 2020 Jul 13;20:336. doi: 10.1186/s12872-020-01616-9 (PMC7359510; doi:10.1186/s12872-020-01616-9)

**Fig 3.** Overview of the study design. The pattern of low-density lipoprotein cholesterol (LDL-C) measurements and lipid-lowering therapy (LLT) prescription redemptions prior to index hospitalization, during index hospitalization, at 6-months follow-up and at 12-months-follow-up among patients with a first-ever diagnosis of ACS.


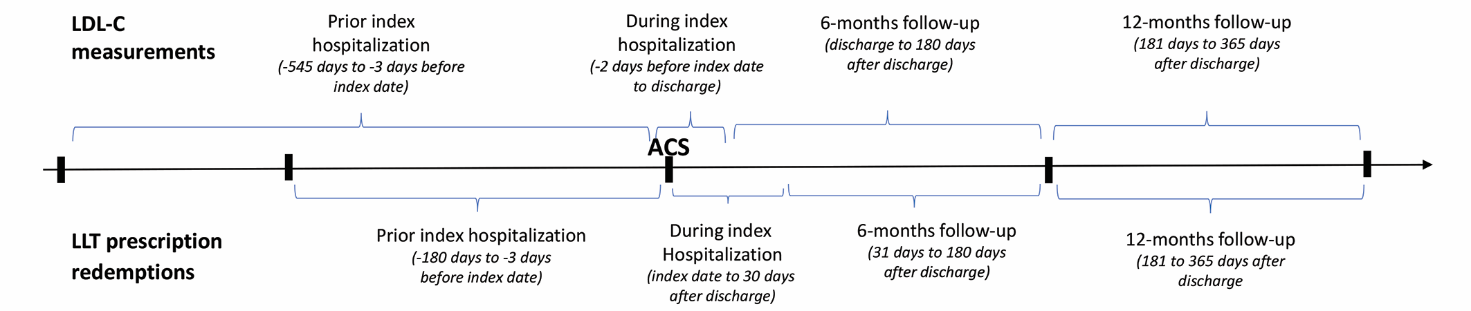

Supplement: Supplementary file 2 — Additional file 2. [file 12872_2020_1616_MOESM2_ESM.docx]
